# Hierarchical Zeolites Containing Vanadium or Tantalum and Their Application in Cyclohexene Epoxidation Reaction

**DOI:** 10.3390/ma16155383

**Published:** 2023-07-31

**Authors:** Paulina Szczyglewska, Agnieszka Feliczak-Guzik, Sylwia Chałupniczak, Izabela Nowak

**Affiliations:** Faculty of Chemistry, Adam Mickiewicz University in Poznań, Uniwersytetu Poznańskiego 8, 61-614 Poznań, Poland; paulina.debek@amu.edu.pl (P.S.); sylwia.jarmolinska@amu.edu.pl (S.C.)

**Keywords:** epoxidation, tantalum, vanadium, hierarchical zeolites, cis-1,2-cyclohexanediol

## Abstract

The aim of this study was the synthesis, characterization, and catalytic application of new hierarchical materials modified with tantalum and vanadium ions. These materials exhibit secondary porosity, thus allowing the reactant molecules to access the active sites of the material while maintaining the acidity and crystallinity of the zeolites. Based on the results, these systems were found to be highly active and selective in the oxidation of cyclohexene. The performance of the catalysts was compared in oxidation processes carried out by conventional and microwave-assisted methods. Microwave-assisted experiments showed that in the presence of a hierarchical FAU zeolite containing Ta, long reaction times could be shortened with increased activity and selectivity under the same residual experimental conditions.

## 1. Introduction

Oxidation catalysis is a very important field of chemical research [1,2]. Oxidation reactions play a key role in the industry as evidenced by the fact that oxidation is the second largest process after polymerization and contributes to 30% of the total production in the chemical industry. Many key chemicals and intermediates such as alcohols, epoxides, aldehydes, ketones and organic acids are produced by selective oxidative catalysis [3]. Among the many processes based on oxidative catalysis, epoxidation of olefins has received special attention. This process is one of the main routes to the production of the above-mentioned groups of compounds, both on laboratory and industrial scales. These compounds are highly useful intermediates for the production of a number of important commercial products such as epoxy resins, paints, surfactants, drugs, food additives, and even agrochemicals [4,5,6,7,8]. Chemical compounds belonging to the epoxy group are intermediates in many organic syntheses. For example, propylene oxide is one of the most important synthetic intermediates produced in industry. In 2009, the production of propylene oxide exceeded 10 million tons per year and this number increased in the following years. Other equally well-known examples are styrene oxide, which is an intermediate in production of pharmaceuticals, dyes, flavors, and nutritional additives [6], and butene oxide, whose annual production exceeds 70,000 tons [5]. Selective synthesis of epoxides is of considerable academic and industrial interest [9]. Traditional epoxidation processes mainly include non-catalytic processes based on the use of chlorine (chlorohydrin processes) and catalytic processes based on organic peroxides and peroxyacids [4,5]. The aforementioned processes have inherent disadvantages from the economic standpoint because they are inherently very capital intensive. Chlorine-based processes primarily have an adverse impact on the environment due to the high production of chlorine-contaminated wastewater. The use of peroxyacids, on the other hand, is of considerable concern because it is not a clean method—an equivalent amount of acidic waste is produced following its use [4]. Nevertheless, the use of oxygen, organic peroxides, and peroxyacids for the direct oxidation of alkenes is a major method for industrial applications. Thus, there is a strong need to develop new epoxidation methods that use safer oxidants and produce little waste [9].

For example, using dihydrogen peroxide as an oxidant is an attractive option for both environmental and economic reasons. This compound is environmentally benign, inexpensive, readily available, water is formed as the only by-product and is a source of high number of active oxygen species [1,5,9]. Dihydrogen peroxide is probably the best terminal oxidant after oxygen and its attractiveness is also due to its good solubility in water and many organic solvents [1]. Under certain circumstances, dihydrogen peroxide is even better than oxygen, insofar as O_2_/organic systems can sometimes spontaneously combust. As a result, epoxidation systems that utilize dihydrogen peroxide in combination with catalytic amounts of inexpensive, relatively nontoxic metals are potentially viable for the production of inexpensive large-scale products and for specialized research applications [10]. Dihydrogen peroxide is a fairly widely studied oxidant that can epoxidize olefinic compounds in the presence of various transition metal-containing catalysts such as Ti, V, Cr, and Mo [4]. Many catalytic systems based on W, Mn or Re have also been reported in epoxidation reactions of a wide range of alkenes using dihydrogen peroxide [9]. Another desirable aspect in the discussed process is the use of heterogeneous catalysts that can be easily separated, regenerated, and reused. Therefore, the development of heterogeneous catalytic processes for epoxidation using H_2_O_2_ as oxidant is very challenging. In this aspect, the development of solid and recyclable catalysts with high performance is a key issue [4].

Heterogeneous catalysts compared to homogeneous catalysts offer the advantages of easy separation from the reaction environment and sometimes higher selectivity, but most of these catalysts suffer from lower activity and instability in epoxidation systems [11]. Titanium-containing silicates, including amorphous titanium-silica materials and Ti-substituted molecular sieves, are the most efficient heterogeneous catalysts in epoxidation reactions. A well-known solid epoxidation catalyst is TS-1 molecular sieve, which was designed as a selective oxidation catalyst operating under mild conditions with H_2_O_2_ as oxidant. The described catalytic system belongs to the zeolite group and contains quaternary titanium centers in microporous silica frameworks [10,12]. In general, fast reaction times with high selectivity (>90%) are achievable but only for simple alkenes [6,9]. Following the success in using TS-1 for the oxidation of various organic compounds with dihydrogen peroxide, titanium catalysts have been extensively studied to overcome the size limitations imposed by the microporosity of TS-1 [6,13], whereby the relatively small pore size of about 0.55 nm, reactions of larger substrates are precluded [10]. To overcome the spatial limitations of TS-1, which hinder the oxidation of substrates, resulting in long reaction times and rather low conversions, other mesoporous and macroporous titanium-containing materials have been developed [9,10]. Therefore, a Ti-MCM-41 catalytic system with larger pore sizes in the meso dimension was designed. The main difference between TS-1 and Ti-MCM-41 is that titanium is located on the surface of Ti-MCM-41, thus circumventing the limitations of small pore size and overcoming the low activity observed for TS-1 with large substrates. However, the lack of synthetic control means that the active sites in Ti-MCM-41 are not uniform, which subsequently leads to reduced selectivity. Furthermore, both catalytic systems suffer from miscibility problems with most substrates in aqueous H_2_O_2_ media. In the case of Ti-MCM-41, the aqueous H_2_O_2_ centers also promote metal oxide agglomeration, which leads to the loss of activity. Therefore, research is ongoing to develop new environmentally friendly olefin epoxidation catalysts [6,14,15,16].

Transition metals play an extremely important role as catalysts in inorganic synthesis. Recently, transition metals, due to their undoubted advantages, have been increasingly used in organic catalytic reactions. Their important feature is that they are relatively biocompatible and a number of chemicals used in everyday life are produced using them. Vanadium and tantalum are attracting increasing attention from scientists, including ours. Vanadium is a relatively non-toxic and inexpensive element thanks to which it plays an important role in biochemistry and medicinal chemistry and its diversity in coordination chemistry has also led to applications in synthesis and catalysis [17]. Tantalum, on the other hand, is an element very similar to niobium in terms of its physicochemical properties—these elements were thought to be one element when they were discovered in the early 19th century. According to the literature data, niobium is a widely used metal in catalytic reactions, so high hopes are placed in tantalum and the recent literature reviews clearly illustrate the progress of this element in catalysis [17]. Given the above information, we decided to investigate the catalytic activity of tantalum and vanadium in the epoxidation reaction of cyclohexene.

In order to overcome the drawbacks of the catalytic systems described above, work has begun on the synthesis of zeolites with hierarchical porous structures that exhibit secondary porosity, i.e., show the presence of at least one additional pore system, mainly in the mesopore range (pore size according to IUPAC from 2 nm to 50 nm) [18].

This arrangement is intended to facilitate the access of larger reagent molecules to the active centers of the material, while maintaining the acidity and crystallinity of the zeolites [19,20]. In turn, the shortening of the diffusion path length due to the reduction in crystallite size results in increased catalyst lifetime [21]. Mesoporous zeolites can positively influence many catalytic reactions in two ways. These hierarchical materials have the potential to increase the number of reactions in which zeolites are used by allowing mass transport of larger reactants and products and by allowing reactions catalyzed by strong acidity to occur on the mesopore surface and at the pore mouth. Alternatively, mesoporous zeolites may simply do a service by improving existing reactions/processes using zeolite catalysts. A summary of the major trends observed for hierarchical zeolites classified by reaction type is shown in Table 1 [22,23]. However, obtaining hierarchical zeolites with secondary porosity is still a challenge for researchers. New and more efficient methods are being developed to synthesize microporous and mesoporous materials. Moreover, such materials should exhibit the properties of zeolites, i.e., additional porosity should not be introduced to the detriment of the microporous structure [19].

**Figure 1:**
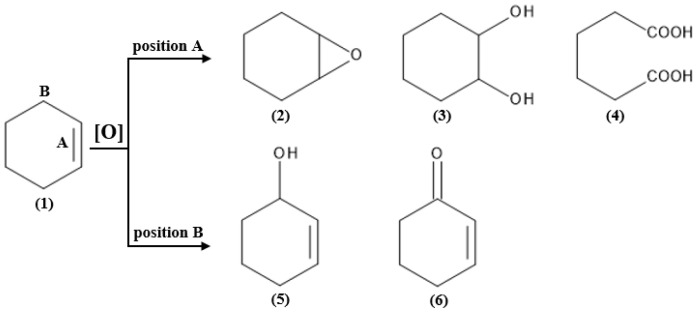
Cyclohexene oxidation reaction pathways: (1) cyclohexene, (2) 1,2-epoxycyclohexane, (3) trans/cis-1,2-cyclohexanediol, (4) adipic acid, (5) cyclohex-2-en-1-ol, (6) cyclohex-2-en-1-one. Adapted with permission from Ref. [27]. Copyright year: 2023, copyright owner’s name: Elsevier, License number: 5598810144247.

As evident from much literature data, 1,2-epoxycyclohexane is an important organic intermediate compound, and therefore, much effort has been devoted to the development of new active and selective catalysts for the epoxidation of cyclohexene that bypass side reactions and hence the formation of large amounts of side products [24]. Since not only 1,2-epoxycyclohexane is a useful intermediate formed in the epoxidation reaction of cyclohexene, selective oxidation of this chemical is very valuable. Other valuable products of cyclohexene epoxidation are 1,2-diols. These chemical compounds are widely used as intermediates in the perfume industry and also in the synthesis of drugs and lubricants [25]. The cis form of this chemical compound is an important organic intermediate—it has two hydroxyl groups, which makes it widely used in the production of polyester resins, epoxy resins, catechol, and also many other products [26]. Therefore, this topic has attracted the interest of synthetic and industrial chemists worldwide, and as a result, there have been many reports on the selective catalytic oxidation of cyclohexene using dihydrogen peroxide in recent years [27]. The described chemical compound is a cheap, abundant and readily available feedstock that is mainly produced by selective hydrogenation of benzene [28,29]. Despite its simple chemical structure, there are two potential oxidation sites in cyclohexene (labeled as site A and site B in Figure 1), and oxidation reactions usually lead to a mixture of products with different oxidation levels and functional groups. Oxidation at the C=C bond site (site A) can lead to 1,2-epoxycyclohexane, trans/cis-1,2-cyclohexanediol, or adipic acid. In contrast, oxidation at the allylic C-H position (site B) can lead to cyclohex-2-en-1-ol or cyclohex-2-en-1-one [27]. The formation of a specific product of cyclohexene oxidation requires different process conditions, and so, in addition to the chemical compounds indicated in Figure 1, the end products of the cyclohexene oxidation reaction can also be cyclohexanol and cyclohexanone [30].

However, several side reactions can occur in epoxidation of alkenes, such as oxidation at allylic positions, epoxide ring opening by hydrolysis or solvolysis, epoxide rearrangement, or even complete decomposition of the C=C double bond. For cyclohexene epoxidation the side reactions of allylic oxidation and epoxide ring opening are particularly disturbing [13]. In summary, the selectivity of the cyclohexene oxidation reaction can be affected by the catalyst and reaction conditions, such as solvent, temperature, catalyst loading, and oxidant. By evaluating these parameters and optimizing the reaction conditions, the oxidation of cyclohexene at its allylic C-H site or C=C bond site can be controlled, which would lead to the selective production of 1,2-epoxycyclohexane, trans/cis-1,2-cyclohexanediol, adipic acid, cyclohex-2-en-1-ol, or cyclohex-2-en-1-one [27].

Accordingly, our research group undertook an experiment whose idea focused on four aspects: (i) synthesis of hierarchical zeolites containing transition metal atoms in their structure, (ii) broad physicochemical characterization of the synthesized catalytic systems, (iii) application of the produced catalysts in the epoxidation of cyclohexene using hydrogen peroxide as an oxidant, (iv) carrying out the epoxidation of cyclohexene in a microwave system (to minimize time and energy) and in a conventional system.

## 2. Materials and Methods

### 2.1. Materials

All materials and chemicals used were of analytical purity and were used as purchased without further purification. The chemicals used to synthesize hierarchical zeolites were: zeolite FAU (Alpha-Aesar, Haverhill, MA, USA), cetyltrimethylammonium bromide—CTABr (Aldrich, Poznan, Poland), tetraethyl orthosilicate—TEOS (Sigma-Aldrich, Poznan, Poland), ammonia (Stanlab, Lublin, Poland). The chemicals used to modify hierarchical zeolites were: vanadium(III) chloride (99%, Merck), tantalum(V) oxide (99.9%, Chempur). The chemicals used to perform the epoxidation reaction were: cyclohexene (Sigma-Aldrich, Poznan, Poland), acetonitrile (Honeywell, Charlotte, NC, USA), dihydrogen peroxide (POCH, Gliwice, Poland).

### 2.2. Catalyst Synthesis

A portion of 0.5 g of hydrogen form of zeolite Y (H,Na-Y, hydrated; (Na)_9_(H_2_O)_x_| [Al_54_Si_138_O_384_]-FAU; mass percent composition of zeolite FAU is provided in Table S1; SiO_2_:Al_2_O_3_ = 5.1:1 mole ratio) was dispersed, using an ultrasonic bath for 30 min at 65 °C, in a mixture containing cetyltrimethylammonium bromide (0.35 g), water (100 g), ethanol (60 g), and ammonia (1.25 g). Further, tetraethyl orthosilicate (0.56 g) and tantalum (Si/Ta = 32) or vanadium source (Si/V = 32), which were tantalum(V) oxide and vanadium(III) chloride, respectively, were added. The whole mixture was then stirred for 4 h at 65 °C. Cetyltrimethylammonium bromide (a structuring agent) was removed at 550 °C for 5 h by calcination. Samples will be further designated as HZ-V and HZ-Ta for hierarchical zeolite FAU containing V or Ta.

### 2.3. Characterization of the Obtained Materials

All of the prepared materials were characterized by using X-ray diffraction (XRD), N_2_ physisorption, transition electron microscopy (TEM), scanning electron microscopy (SEM), scanning electron microscopy with elemental microanalysis with EDX (SEM/EDX), and X-ray photoelectron spectroscopy (XPS).

XRD patterns were measured with a Bruker AXS DB Advance diffractometer (CuKα radiation, λ = 0.154 nm) in two 2Q ranges: 0.6–8° (small angle XRD (SXRD)) and 4–60° (wide angle XRD (WXRD)) with the step size of 0.02° or 0.05°. XRD patterns in the wide angle range were indexed in the cubic symmetry (space group Fd-3m) and lattice parameters (a_0_) were determined.

Adsorption/desorption experiments using nitrogen as adsorbent were carried out at −196 °C on a Autosorb-iQ, Quantachrome. Before each measurement, the samples were first outgassed at 300 °C for 3.5 h in vacuum. The N_2_ isotherms were used to determine the specific surface areas using the standard BET (Brunauer-Emmett-Teller) equation in the relative pressure (p/p_0_) from 0.05 to 0.2 and the cross-sectional area of nitrogen molecule of 0.162 nm^2^. Pore sizes were obtained from the N_2_ adsorption branch, using BJH method (Barrett-Joyner-Halenda) with the corrected Kelvin equation, i.e., KJS-BJH method at the maximum of pore size distribution. The single-point pore volume was obtained by conversion of the volume adsorbed at p/p_o_ = 0.99 to the volume of liquid nitrogen. The volume of micropores was calculated by α_s_-method.

Transmission electron microscopy images were obtained using a JEOL-2000 transmission electron microscope with an accelerating voltage of 80 kV. Scanning electron microscopy images were obtained on a Zeiss EVO-40 apparatus working at the voltage of 19 kV.

The morphological and structural features of the prepared catalysts were investigated by scanning electron microscopy (LEO Electron Microscopy Ltd., 1430 VP England) coupled with the energy dispersive X-Ray detector with 20 keV accelerating voltage.

X-ray photoelectron spectroscopy measurements were performed with a Specs photoelectron spectrometer equipped with a monochromatic microfocused AlKα X-ray source (1486.7 eV). The power of the X-ray source was 150 W. 

Determination of the nature of the chemical bonds of the elements in the sample was made using the CasaXPS 2.3.22 program. A Shirley-type baseline was used to cut off the background, while the signals were decomposed into mixed Gauss and Lorentz lines. The binding energy scale of the spectra was shifted to 284.8 eV, which is assigned to the signal corresponding to C-C (adventitious carbon) bonds. Identification of chemical states of the analyzed elements, based on the obtained spectra, was carried out on the basis of data from databases and scientific publications.

The light absorption properties of HZ materials were determined by ultraviolet (UV)–visible spectrum (Cary 500)—DR-UV-Vis study.

### 2.4. Cyclohexene Epoxidation Reaction

Before carrying out the epoxidation reaction, the catalysts were subjected to activation. For this purpose, they were placed in an oven (Nabertherm, New Castle, DE, USA) at 400 °C for 2 h. The catalyst (0.02 g), cyclohexene (0.1 mL), acetonitrile (5 mL), and dihydrogen peroxide (0.085 mL) were placed in a suitable glass reaction vessel with a magnetic stirrer inside.

#### 2.4.1. Microwave Method

The vessel with the prepared reaction mixture was located in a microwave reactor (Discovery System CEM). The reactions were carried out at two temperatures, viz 45 °C or 65 °C for 6 h (sampled every 1 h). The reaction mixtures were filtered to separate the liquid products from the solid catalyst and then transferred to 1.5 mL vials for chromatographic analysis.

#### 2.4.2. Conventional Method

The vessel with the prepared reaction mixture was placed on a magnetic stirrer equipped with a thermocouple controlling the reaction temperature (Ika-Werke RCT classic). Reactions were carried out at 45 °C for 48 h (sampled after: 2 h, 4 h, 6 h, 8 h, 12 h, 24 h, 48 h). The reaction mixtures were filtered to separate the liquid products from the solid catalyst and then transferred to 1.5 mL vials for chromatographic analysis.

### 2.5. Chromatographic Analysis

Gas chromatograph with a flame ionization detector (VARIAN 3900 GC, capillary column CPWAX57CB: length 25 m, diameter 0.32 mm, film thickness 1.2 µm) and a gas chromatograph with a mass detector (VARIAN 4000 GC, capillary column CPWAX57CB: length 25 m, diameter 0.32 mm, film thickness 1.2 µm) were used for this purpose. Identification of reaction products was performer in two ways: on the basis of the retention times of the test compounds and on the basis of the retention times of commercially available standards and using the NIST compound library.

## 3. Results and Discussion

### 3.1. Catalyst Characterization

**Figure 2:**
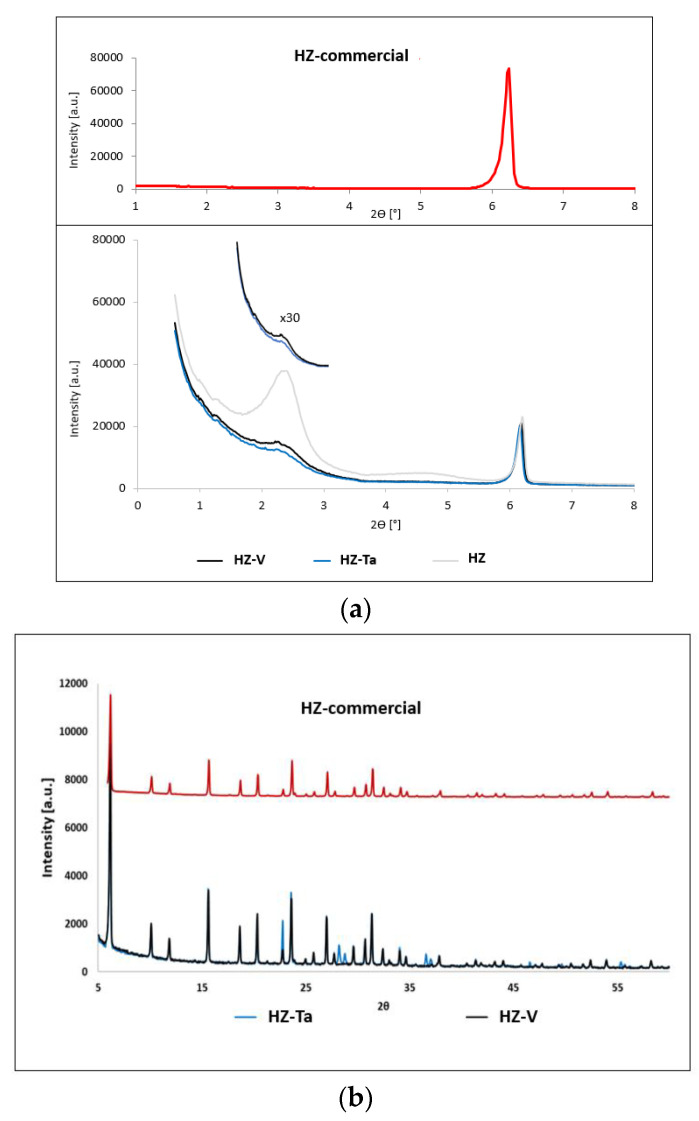
XRD patterns of hierarchical zeolites: (a) low-angle range; (b) wide-angle range.

Diffractograms of commercial zeolite FAU (HZ-commercial) and hierarchical zeolites modified with tantalum and vanadium ions are shown in Figure 2.

The diffractograms of the synthesized hierarchical materials based on commercial zeolite, in the low-angle range (Figure 2a), are characterized by the occurrence of an additional reflection at an angle of 2θ ~ 2.5°. The presence of one very intense diffraction peak, d_100_, was characterized as a mesoporous structure; however, the broadened peak indicates incorporation of randomness in a structure (symmetry cannot be determined) [31]. These results indicate the acquisition of an additional mesoporous structure. Additionally, wide-angle diffractograms (Figure 2b) of the obtained hierarchical materials confirm the structure preservation of the commercial zeolite. Difference in the scattering power which is specific to each cation and also by a slightly different sites occupation in the pores caused slight changes in the unit cell parameter (Table 2). The highest change was observed during hierarchization process [32]. Moreover, distinct diffraction peaks were observed for the HZ-Ta material at 2θ = 22.70°, 28.19°, 28.75°, 36.59° and 36.6°. The observed diffraction peaks can be attributed to Ta_2_O_5_ (hexagonal phase) [33].

**Figure 3:**
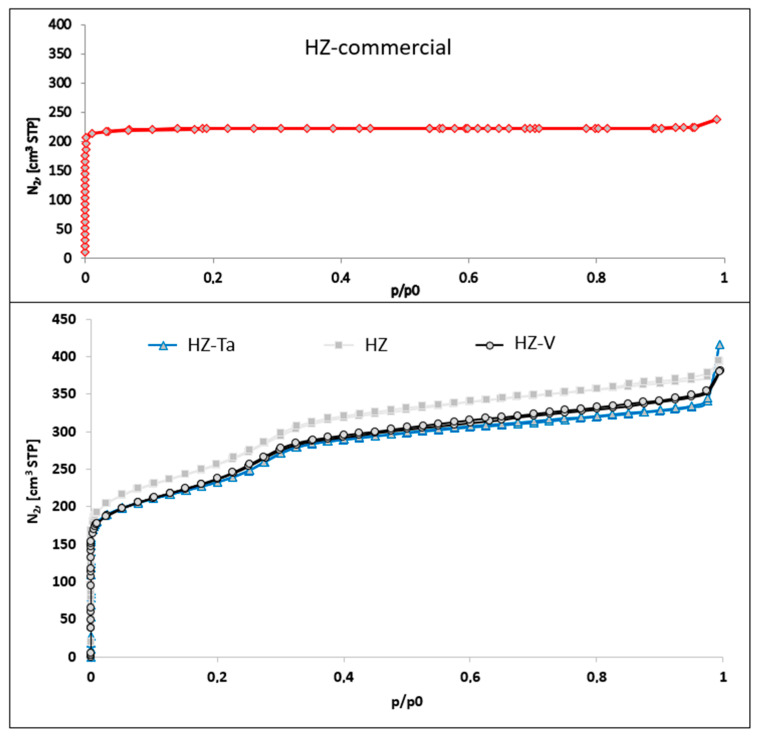
N_2_ adsorption isotherms of hierarchical zeolites.

Nitrogen adsorption/desorption isotherms for hierarchical materials modified with tantalum or vanadium ions and commercial zeolite are shown in Figure 3. The isotherm obtained for the commercial zeolite used to synthesize the hierarchical materials is of type I (according to IUPAC classification). This type of isotherm is characteristic of microporous materials [34]. On the other hand, isotherms obtained for materials modified with tantalum ions and vanadium are a mixture of type I and type IV. Type IV according to the IUPAC classification is characteristic of mesoporous materials [34].

From the analysis of the measured isotherms, both micropores, which are associated with zeolite crystals (primary porosity), and mesopores (secondary porosity) were found. These pores are generated during synthesis assisted by the addition of a structuring agent, CTABr, and a silicon source, TEOS [35]. The obtained shape of the isotherms may suggest the presence of modified zeolite crystallites with wedge pore type [34].

Table 2 shows the structural/textural parameters of the synthesized hierarchical materials. The specific surface area of the materials was determined using the BET method. The S_BET_ specific surface areas of the modified materials, relative to commercial microporous zeolite HZ, increase for all materials studied. When the material is modified with the corresponding metal ions, the surface areas decrease, which may be due to pore blocking by the metal nanoparticles i.e., tantalum or vanadium.

The pore volume distribution function was calculated on the basis of the KJS-BJH algorithm. All materials, except the commercial zeolite, have a uniform pore size distribution in the mesopore range. Micro- and mesopore volumes were determined using the α_s_-plot method. A higher micropore volume was observed for the commercial material compared to the hierarchical materials synthesized and follows the order HZ-commercial > HZ > modified with Ta or V HZ. All the synthesized materials have mesopore volume (V_tot_-V_micro_) larger than the commercial zeolite. The introduction of V and Ta caused a small decrease in all parameters studied with the associated increase in outer surface area, indicating that additional porosity was obtained. Pore size distribution is shown in the Supplementary Materials (Figure S1).

**Figure 4:**
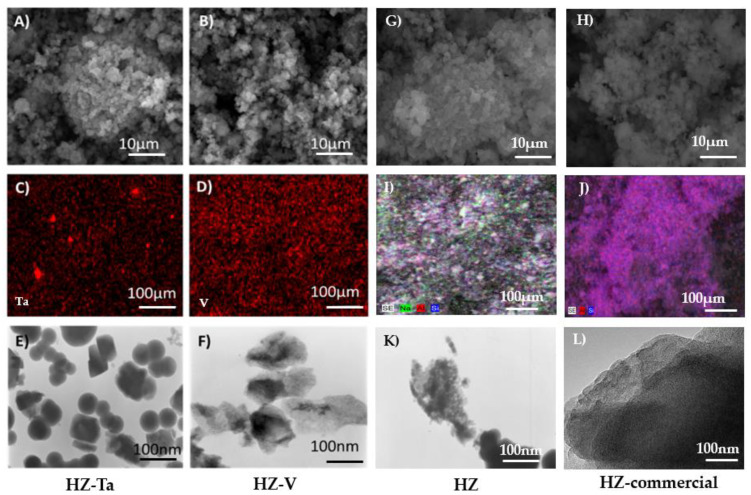
Images of hierarchical zeolites: (**A**,**B**,**G**,**H**)—SEM, (**C**,**D**,**I**,**J**)—SEM-EDX, (**E**,**F**,**K**,**L**)—TEM.

Figure 4 shows the images taken by TEM, SEM, and SEM/EDX analysis for the materials modified with tantalum and vanadium ions. TEM images of both tantalum and vanadium ion modified materials suggest the presence of tantalum oxide and vanadium oxide nanoparticles on their surface. These nanoparticles (most probably tantalum(V) oxide and vanadium(V) oxide) appear as clustered crystallites of irregular shape. 

On the other hand, the images taken by SEM indicate the presence of aggregates of nanocrystallites. The SEM/EDX analysis indicates an uneven distribution of the introduced metal ions, i.e., tantalum and vanadium. EDX profiles are shown in the Supplementary Materials (Figures S2–S5). The calculated ratio of Si/T was between 20 and 100 and higher for Ta (~20) than for V (~100). The previous studies based on DFT calculations on zeolites containing group 5 elements have shown that in the case of vanadium, most sites prefer to accommodate a V = O group, whereas for tantalum, a preference for the Ta–OH group is observed [36]. This can explain why the content of tantalum turned out to be five times higher than that of vanadium. The geometry and stability of the tetrahedral T species (V or Ta) with T = O group and pentacoordinated T species with T–OH groups play important role in the localization of metals in the bulk structure. It is worthy to stress that EDX measures 300 nm to 5000 nm below the surface. For the catalysis more important is content of metals localized on the surface (XPS) than the bulk one (EDX)

HZ-Ta and HZ-V were analyzed by XPS in the BE (binding energy, eV) regions of Si 2p, O 1s, C 1s, Ta 4f and V 2p.

**Figure 5:**
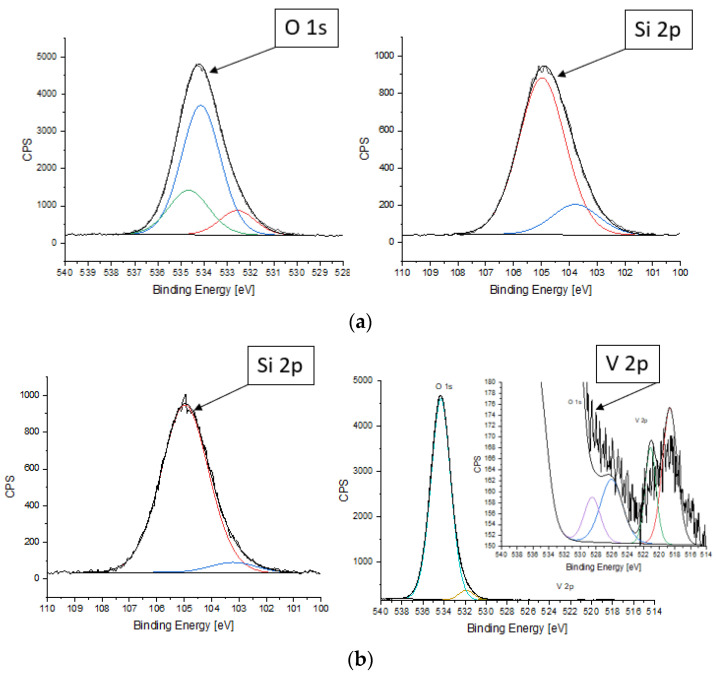
XPS spectra of HZ-Ta (**a**) and HZ-V (**b**) samples.

XPS analysis makes it possible to analyze the presence of an element in a surface of sample (measures only the top 10 nm) and determine its chemical state (Figure 5). No Ta 4f photoelectron signal was observed on XPS spectrum due to the fact that the O 2s photoelectron signal overlaps with Ta 4f photoelectron signal [37]. The Ta 4d photoelectron signal, which would have appeared if this element had been present on the sample surface, was not observed. Thus, the presence of tantalum ions on the sample surface using this technique is not possible. This may indicate a strong dispersion of tantalum on the surface of the material. For samples with such properties, the XPS method is insensitive. This may suggest that the penetration of pores by tantalum ions was very effective since the SEM/EDX results confirm the presence of tantalum ions.

For HZ-V, as a result of spin-orbit cleavage, two doublets, designated V 2p_3/2_ and V 2p_1/2_, were obtained with an intensity ratio of 2:1 (FWHM = 2.2 eV was used). The doublet of V 2p_3/2_ and V 2p_1/2_ with values of 518.7 eV and 526.1 eV, respectively, with a cleavage of 7.4 eV indicates the presence of vanadium V^4+^ ions. The doublet of V 2p_3/2_ and V 2p_1/2_ with values of 521.1 eV and 528.5 eV, respectively, with a cleavage of 7.4 eV indicates the presence of vanadium V^5+^ ions [38]. Thus the content of V^5+^ ions is higher than V^4+^ ions. Additionally, for both samples, the signal representing silicon was split into two components (FWHM = 2.0 eV was used): 103.2 eV—the value corresponding to silicon atoms in chained together or bonded with carbon, hydrogen, oxygen, or nitrogen and 105.0 eV—the value corresponding to silicon atoms in tetrahedral coordination, e.g., SiO_4_, Si-OH, present in zeolite structure [39]. This is due to the fact that detached aluminum species are enriched in the surface region of the zeolite. The signal representing carbon was split into four components (FWHM = 1.6 eV was used): 284.8 eV (C-C); 285.7 eV (C-O-C); 286.8 eV (C=O); 288.9 eV (O-C=O) [34]. It can be seen from the above data that the element is present on the surface of the test sample in the form of airborne contamination. For HZ-Ta, the signal representing oxygen was split into three components (FWHM = 1.8 eV was used): 532.6 eV (O-Al); 534.1 eV (O-Si); 534.7 eV (O-C). On the other hand, for HZ-V, the signal representing oxygen was split into three components (FWHM = 1.8 eV was used): 530.1 eV (O-V); 531.9 eV (O-V/O-Al); 534.3 eV (O-Si) [37,40]. The shifting of binding energy in zeolites depends on charge transfers in the zeolite lattice and the ionicity and covalency of Si-O and Al-O [41,42]. The percentage of elements in the surface layer of the HZ-Ta and HZ-V are shown in the Table 3.

**Figure 6:**
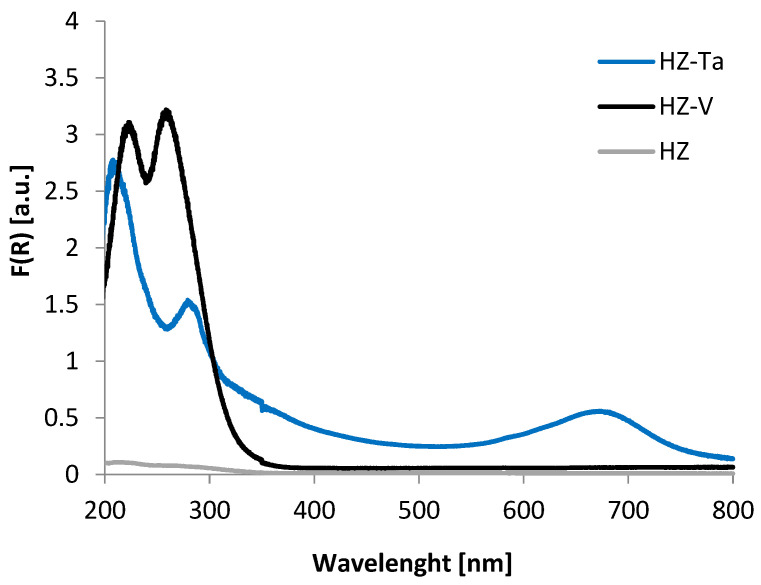
DR UV-Vis spectra for hierarchical zeolites: HZ, HZ-V, HZ-Ta.

The nature and environment of V and Ta species have been studied by DR-UV-Vis spectroscopy (Figure 6). This technique allows distinguishing tetrahedral and octahedral sites. For HZ-Ta, a broad band at 600–800 nm (d-d transition) was observed indicating the presence of octahedral tantalum, typical for Ta_2_O_5_. Additionally, a band at 300 nm appeared resulting from Ta=O or Ta-OH [43]. For the vanadium-containing sample, only tetrahedral species were registered (235 and 265 nm representing oxygen-to-tetrahedral vanadium charge transfer [44]).

### 3.2. Cyclohexene Epoxidation Reaction

#### 3.2.1. Microwave Method

**Figure 7:**
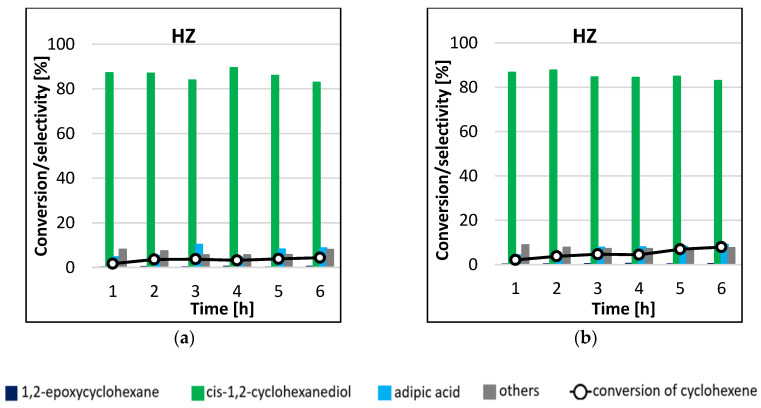
Conversion of cyclohexene and selectivities to individual reaction products using HZ catalyst for the processes carried out at 45 °C (**a**) and 65 °C (**b**) by microwave-assisted method.

The results of HZ catalyst activity in cyclohexene epoxidation reaction, carried out at two temperature variants, are shown in Figure 7. As can be concluded from the analysis of the graphs, the performance of the unmodified catalyst in the cyclohexene epoxidation reaction was low. In both processes carried out, the main reaction product was cis-1,2-cyclohexanediol (selectivity > 80%). However, the conversion of cyclohexene was negligible, so the results obtained should not be compared. The blank test did not give any conversion of cyclohexene after 6 h of reaction duration.

**Figure 8:**
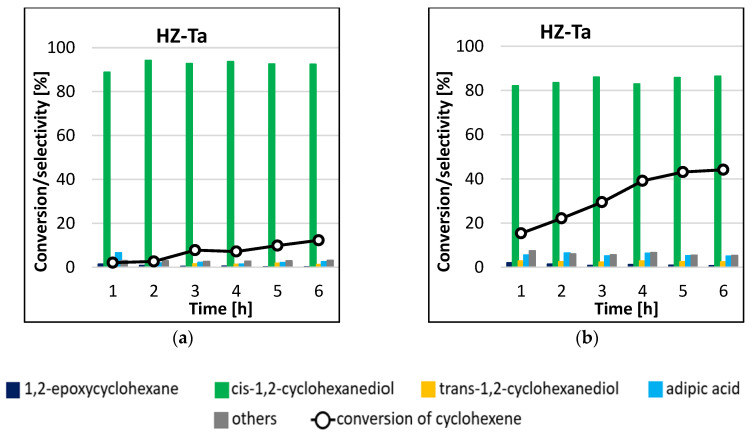
Conversion of cyclohexene and selectivities to individual reaction products using HZ-Ta catalyst for the processes carried out at 45 °C (**a**) and 65 °C (**b**) by the microwave-assisted method.

The results of HZ-Ta catalyst activity in cyclohexene epoxidation reaction are shown in Figure 8. The main product of the reaction in the two temperature variants was cis-1,2-cyclohexanediol. The selectivity to this product ranged from 89–94% and 82–87% for reactions carried out at 45 °C (Figure 8a) and 65 °C (Figure 8b), respectively. Trans-1,2-cyclohexanediol, adipic acid, and 1,2-epoxycyclohexane were also formed in small amounts. The conversion of cyclohexene increased with increasing reaction time, from 15% to 44% for the process carried out at 65 °C. On the basis of the results obtained, it can be concluded that the process temperature had a significant effect on the activity of the tantalum-modified catalyst. It is worth mentioning that the tantalum catalyst proved to be highly selective towards the production of cis-1,2-cyclohexanediol; the selectivity to the mentioned product remained constant as the conversion increased. The blank gave no conversion of cyclohexene after 6 h of reaction duration.

**Figure 9:**
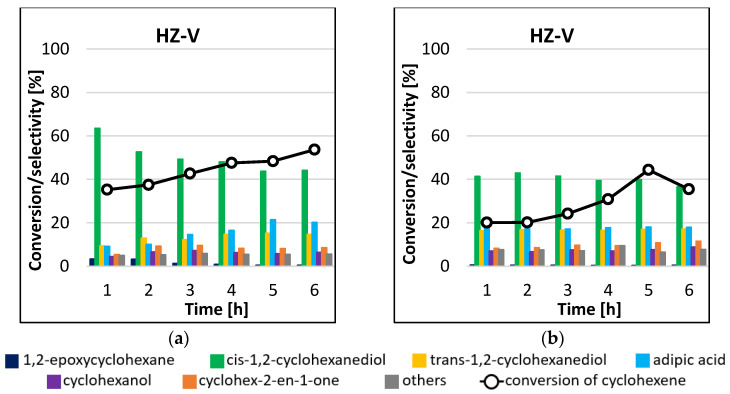
Conversion of cyclohexene and selectivities to individual reaction products using HZ-V catalyst for the processes carried out at 45 °C (**a**) and 65 °C (**b**) by the microwave-assisted method.

Analysis of the results of the cyclohexene epoxidation reactions catalyzed by HZ-V (Figure 9) permits drawing several conclusions. The first is that the product formed in the greatest amounts in the process carried out at 45 °C was cis-1,2-cyclohexanediol, and its amount decreased with increasing cyclohexene conversion (Figure 9a). After 1 h of the reaction, the selectivity to the main reaction product was 64%, the highest value in all experiments. Trans-1,2-cyclohexanediol and adipic acid were also produced in great amounts. The selectivity to these products in favor of increasing amounts of cis-1,2-cyclohexanediol. The formation of cyclohexanol, cyclohex-2-en-1-one, and 1,2-epoxycyclohexane was also observed in amounts less than 10%. For the process carried out at the higher temperature, the main reaction product was also cis-1,2-cyclohexanediol (Figure 9b). The highest conversion of 44% was obtained after 5 h of the process. No effect of the reaction time on the selectivity to individual products was observed. Interestingly, the obtained conversions of cyclohexene were lower than for the process carried out at the lower temperature. A blank test did not give any conversion of cyclohexene after 6 h of reaction.

#### 3.2.2. Conventional Method

**Figure 10:**
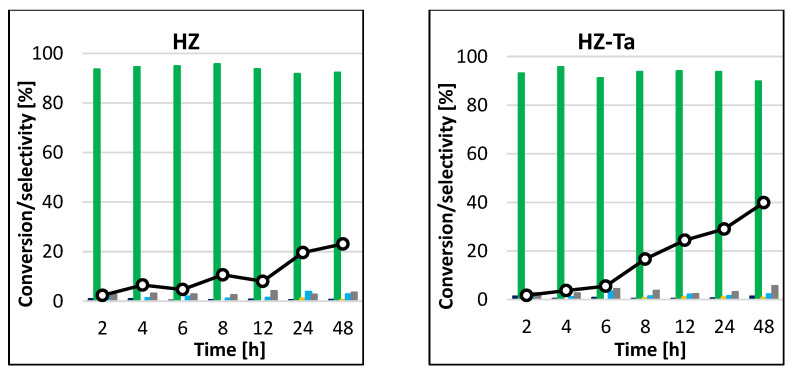

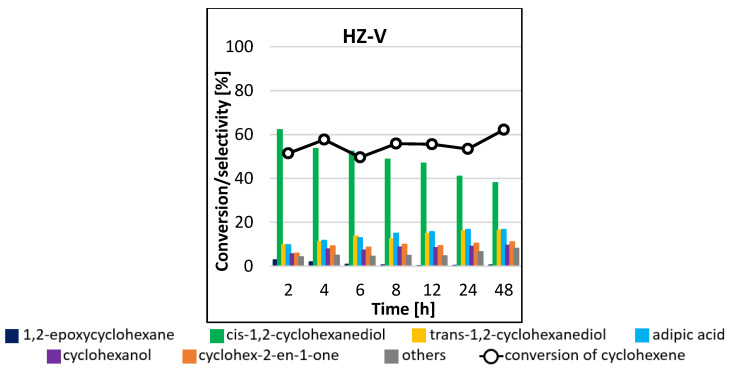
Conversion of cyclohexene and selectivities to individual reaction products using different catalysts for the process carried out at 45 °C by the conventional method.

In general, the epoxidation reaction under conventional heating takes a long time (>24 h) to achieve good quantitative conversion of the starting material. For the non-transition metal atom-modified HZ catalyst, the conversion of cyclohexene increased with increasing reaction time, reaching a peak of 23% after 48 h (Figure 10). The HZ-Ta catalyst had slightly greater activity, as after 48 h, the highest conversion of the test molecules of 40% was obtained. Thus, the presence of tantalum atoms had a positive effect on the conversion of cyclohexene in the epoxidation reaction. In both cases, the catalytic systems proved to be extremely selective to the formation of cis-1,2-cyclohexanediol, with virtually no formation of side products under the reaction conditions tested (selectivity to the main product > 90%). The situation was slightly different when a vanadium-containing catalyst was used—the results obtained were slightly different at each sampling time. Conversion of cyclohexene oscillated between 50 and 62%, while the selectivities to individual reaction products differed only slightly. The highest selectivity was observed to cis-1,2-cyclohexanediol, and it decreased with increasing process time. Formations of trans-1,2-cyclohexanediol, adipic acid, cyclohexanol, and cyclohex-2-en-1-one and traces of 1,2-epoxycyclohexane were also observed. The blank gave no conversion of cyclohexene after 48 h of reaction.

#### 3.2.3. Microwave-Assisted Method vs. Conventional Method

A comparison of the results obtained for the processes carried out under identical conditions in the presence of tantalum-modified HZ-Ta catalyst, in which the only difference was the heating method, leads to some interesting observations. In the reaction, run for 6 h at 45 °C, the conversion of cyclohexene in the microwave system (Figure 8a) was 12%, while in the conventional system it was 6% (Figure 10), so that the use of microwave radiation resulted in an increase in the conversion of the starting substrate by 100%. On the other hand, in the process carried out at 65 °C in the microwave system (Figure 8b), the conversion of cyclohexene reached 44% in 6 h. A comparison of the data obtained for the reactions carried out in the conventional system and in the microwave system at 65 °C indicates that for the process in the former system, the cyclohexene conversion of 40% was obtained after 48 h of the reaction, while the same conversion was obtained already in 4 h of the process carried out in the microwave system. Thus, in this case, by increasing temperature by 20 °C and using the microwave system, the reaction time was reduced by 44 h, with comparable catalyst efficiency. In all variants of the reactions carried out, the main product was cis-1,2-cyclohexanediol, which was formed with a comparable selectivity (>80%).

The results obtained for the analogous processes in the presence of the systems containing vanadium were also analyzed. In the process carried out in the conventional system, the conversion of cyclohexene of the order of 51% was achieved already after 2 h of the reaction (Figure 10). For the process carried out in the microwave system at 45 °C (Figure 9a), a comparable result was obtained in 6 h of the reaction, while for the process carried out at 65 °C (Figure 9b), the conversion did not exceed 50%, reaching a maximum result of 44% in 5 h of reaction. Therefore, the application of microwave radiation to the reaction run in the presence of the vanadium-containing catalytic system did not positively affect its performance. In all cases analyzed, the product formed in the highest amounts was cis-1,2-cyclohexanediol.

#### 3.2.4. Summary of All Experiments Carried out

In general, 1,2-epoxycyclohexane and 1,2-cyclohexanediol are the most common products of cyclohexene epoxidation. As cyclohexene is epoxidized to 1,2-epoxycyclohexane which is further hydrolyzed to 1,2-cyclohexanediol in acidic media. As follows from our results, a large portion of 1,2-epoxycyclohexane was just hydrolyzed to cis-1,2-cyclohexanediol. Several factors are behind this result. One of them may be the structure of the carrier, making it difficult for 1,2-epoxycyclohexane to diffuse to the solution because of the narrow mesopores, which led to a further reaction, i.e., the hydrolysis of epoxide to diol [45]. Moreover, hydrolysis reactions generally require acidic sites of medium strength, and zeolites are known to be among the most widely studied heterogeneous catalysts with Lewis-acid-like centers [46]. Additionally, the introduction of metal atoms generates additional acid centers leading to increased Lewis acidity, catalyzing the ring opening of epoxide to diol and related oxidation products [47,48]. Another factor is the formation of water as a by-product of dihydrogen peroxide-mediated epoxidation of cyclohexene due to the occurrence of hydroperoxy dehydration of the intermediate compound to allylic ketone, which also supports the occurrence of hydrolysis of 1,2-epoxycyclohexane to cis-1,2-cyclohexanediol [49].

The differences between the catalytic systems containing different metals, i.e., Ta or V, may be due to the specific electronic structure of each of these metals. It is well known that the surface properties of the catalyst play an important role in the activity and selectivity of the transition metal atoms deposited on the support. Surface properties affect, among other things, the adsorption of substrates and desorption of products or alter the local coordination and electronic character of the active forms of metals [50]. Poor catalytic activities have been attributed to poisoning of the active sites by coordination of water within them and/or deactivation of the hydroperoxide intermediate. Many catalysts also catalyze the decomposition of dihydrogen peroxide to water and gas oxygen, leading to low catalytic efficiency [50]. According to the results obtained in our study, the conversion of cyclohexene using HZ-V catalyst appeared to be higher than that obtained in the presence of HZ-Ta catalyst. This may also be due to the high dispersion of tantalum atoms and monodispersity, which leads to the exposure of more external surface area of the catalyst. It can be assumed that cyclohexene (nucleophile) would preferable interact with nucleophile. The vanadium species is by far the most electrophile of the three group 5 elements, whereas Ta framework sites are predicted to be the most nuclophile and thus expected to be the most active ones [51]. In our case, we observed the opposite trend due to the presence of water in the reaction media; however, one has to keep in mind that the amount of tantalum incorporated was five times higher than that of vanadium. Thus, further studies are required. Additionally, it was previously shown that textural properties are more important parameters in this kind of reaction, opposite to the gas phase reaction [52].

The support of chemical reactions with microwave radiation is based on the use of microwave dielectric heating. It results from the ability of a given material (catalyst, reactant, solvent) to adsorb the energy assigned to microwave radiation and convert it into thermal energy. The primary difference in heat transfer when heating a material in thermal and microwave fields is that microwave energy, unlike thermal energy, is delivered directly to a large volume, thus avoiding thermal lags associated with conduction and/or convection. Consequently, temperature gradients and excessive heat buildup during thermal processing can be reduced by adjusting the microwave power [53]. The described phenomenon explains the better performance of HZ and HZ-Ta catalyst in the process with microwave heating than with conventional heating.

## 4. Conclusions

In summary, mesoporous hierarchical FAU zeolite materials containing vanadium and tantalum were prepared using quaternary ammonium cations as organic structure directing agents under hydrothermal conditions. Their comprehensive characterization showed that they exhibited good structural and textural parameters, which are typical for materials from this group.

The synthesized materials were used as catalysts in the epoxidation reaction of cyclohexene by microwave and conventional methods. The microwave method did not affect the acceleration of the cyclohexene epoxidation process—the cyclohexene conversion results obtained were comparable. The vanadium catalyst showed slightly better activity. In turn, the tantalum-modified catalyst was more selective towards the production of cis-1,2-cyclohexanediol. These results confirm that the hierarchical zeolite FAU materials containing vanadium or tantalum ions are potentially interesting catalysts for epoxide rearrangement reactions.

As shown in the literature data, catalytic systems containing Ti or Nb are very often used in the epoxidation reaction of cyclohexene. As we have shown through the present study, catalysts modified with Ta or V are also active in the epoxidation of cyclohexene. Moreover, the advantage of using hierarchical zeolites is that there are no size limitations imposed by the microporosity of the zeolites which could preclude reactions of larger substrates. Additionally, the acidity and crystallinity that are characteristic of zeolites are preserved, which affects the type of reaction products formed in the epoxidation of cyclohexene. Moreover, popular catalytic systems based on mesoporous materials (e.g., MCM-41) have problems with miscibility with most substrates in aqueous H_2_O_2_ environments, which does not happen when using catalysts synthesized by us.

## Figures and Tables

**Table 1:** Overview of different types of reactions catalyzed by hierarchical zeolites and main observation, based on [22,23].

Reaction Type	Main Observation
Alkylation reactions	Increased activity
Transalkylation reactions	Improved stability
Isomerization reactions	Higher activity
Cracking of light substrates	No significant improvement
Heavy-base cracking	Significantly higher activity
Aromatization and MTH reactions	Longer catalyst activity
Condensation reactions	Higher activity; higher selectivity for large size products

**Table 2:** Porous structure parameters (textural from nitrogen sorption and structural from XRD) of the materials studied.

Samples	Unit Cell Parameter [nm] *	BET Surface Area [m^2^/g]	Pore Volume [mL/g]		Mesopore Size [nm]
			Total Pore Volume	Micropore Volume	
HZ-commercial	2.467	718	0.37	0.34	-
HZ	2.474	891	0.56	0.16	3.33
HZ-Ta	2.477	839	0.50	0.15	3.30
HZ-V	2.479	823	0.51	0.14	3.33

* data from XRD.

**Table 3:** The percentage of elements in the sample-based XPS analysis.

HZ-Ta	
Elements	Percentage in the Samples [%]
Oxygen	52.80
Silicon	35.48
Carbon	10.14
Aluminum	1.58
**HZ-V**	
Oxygen	57.12
Silicon	38.83
Carbon	3.17
Aluminum	0.63
Vanadium—total amount in the sample	0.24 (65.78% - V^4+^ and 34.22% V^5+^)

## Data Availability

Data will be made available upon reasonable request.

## Supplementary Materials

The following supporting information can be downloaded at: https://www.mdpi.com/article/10.3390/ma16155383/s1, Figure S1: Pore size distribution of hierarchical materials; Figure S2: EDX profile for HZ-commercial; Figure S3: EDX profile for HZ; Figure S4: EDX profile for HZ-Ta; Figure S5: EDX profile for HZ-V; Table S1: Chemical composition of zeolite Y (FAU).
