# Supplementary material for: Hierarchical Zeolites Containing Vanadium or Tantalum and Their Application in Cyclohexene Epoxidation Reaction
Source: Materials (Basel). 2023 Jul 31;16(15):5383. doi: 10.3390/ma16155383 (PMC10419380; doi:10.3390/ma16155383)
Supplement: Supplementary file 1 [file materials-16-05383-s001.zip › materials-2456570-supplementary.pdf]

Hierarchical Zeolites Containing Vanadium or Tantalum and Their Application in Cyclohexene Epoxidation Reaction

Paulina Szczyglewska, Agnieszka Feliczak-Guzik *, Sylwia Chałupniczak and Izabela Nowak *

Faculty of Chemistry, Adam Mickiewicz University in Poznań, Uniwersytetu Poznańskiego 8, 61-614 Poznań, Poland; paulina.debek@amu.edu.pl (P.S.); sylwia.chałupniczak@amu.edu.pl (S.C.)

* Correspondence: agaguzik@amu.edu.pl (A.F.-G.); nowakiza@amu.edu.pl (I.N.); Tel.: +48-61-829-1747 (A.F.-G.); +48-61-829-1580 (I.N.)

HZ-commercial

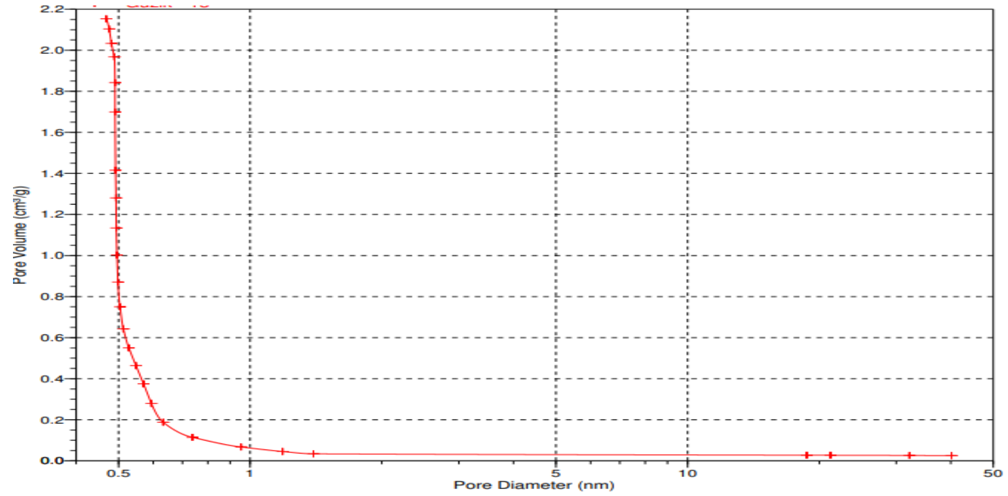

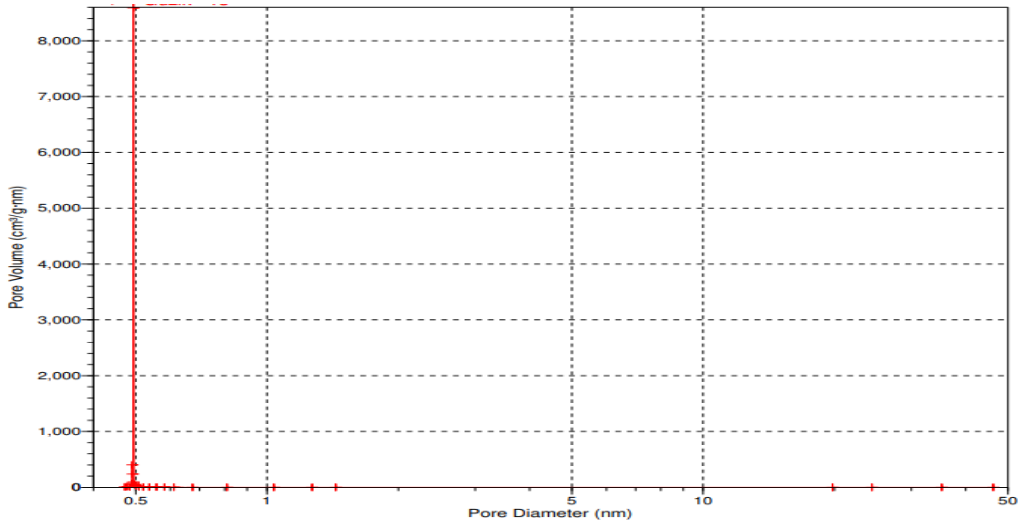

HZ

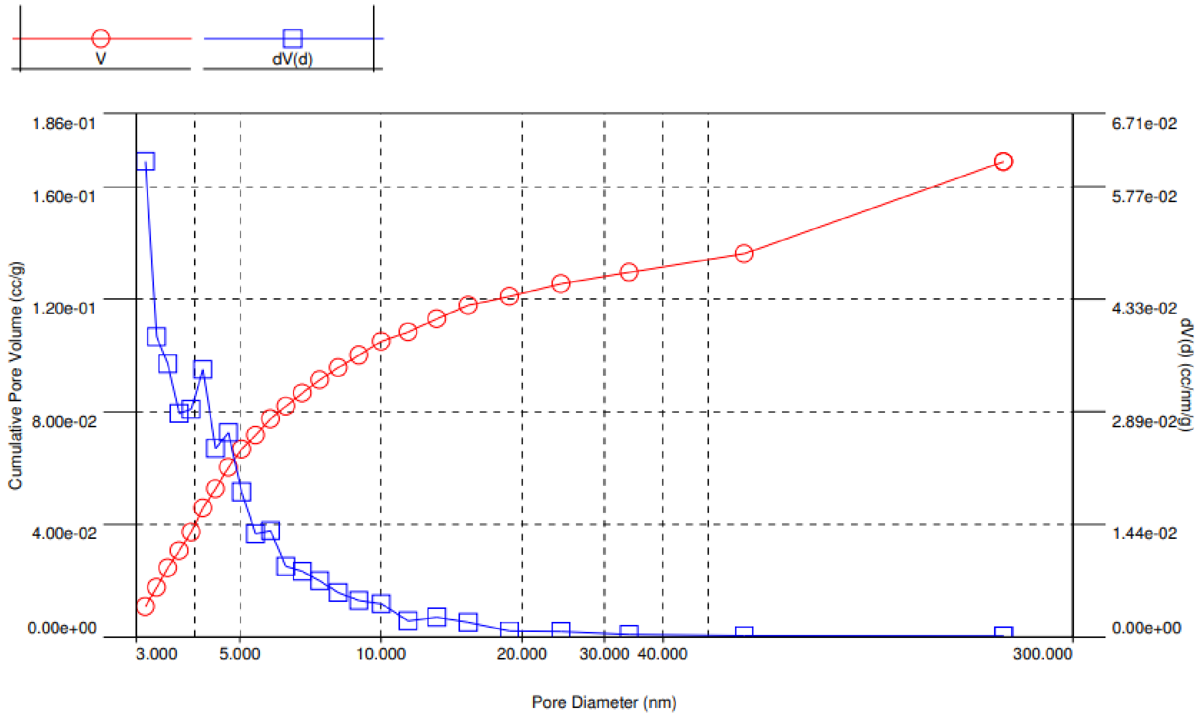

V
dV(d)
1.86e-01
1.60e-01
1.20e-01
8.00e-02
4.00e-02
0.00e+00
Cumulative Pore Volume (cc/g)
6.71e-02
5.77e-02
4.33e-02
2.89e-02
1.44e-02
0.00e+00
dV(d) (cc/nm/g)
3.000
5.000
10.000
20.000
30.000
40.000
300.000
Pore Diameter (nm)

HZ-Ta

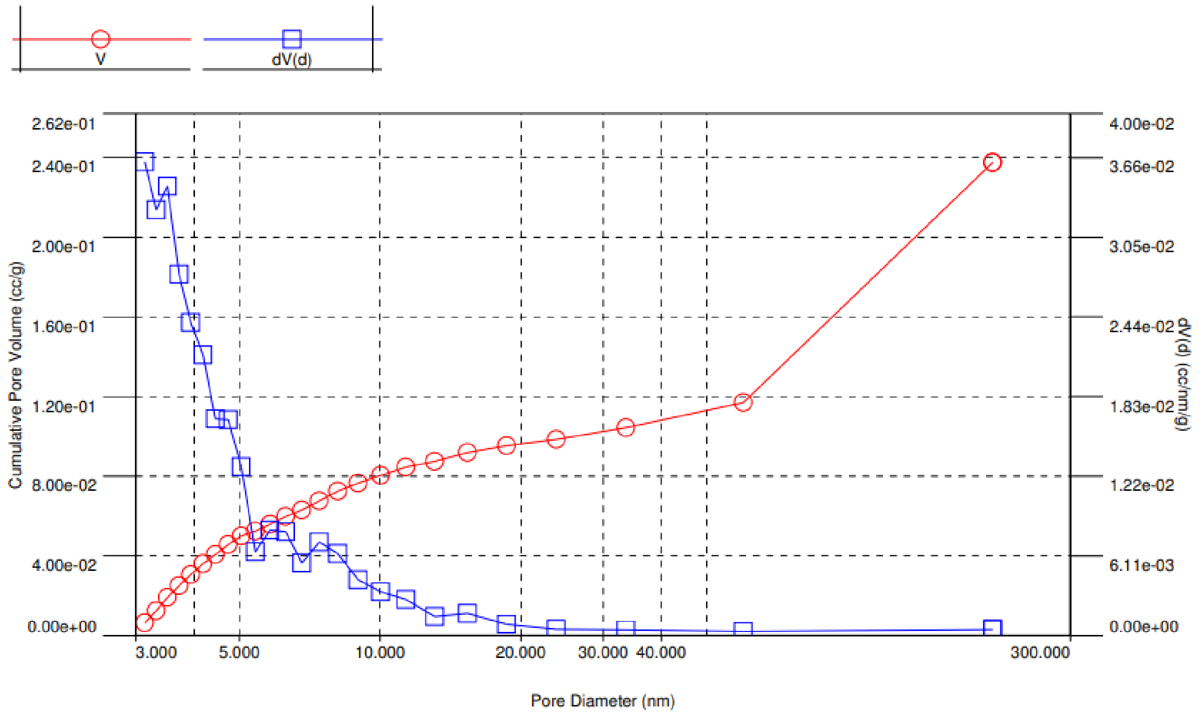

V
dV(d)
2.62e-01
2.40e-01
2.00e-01
1.60e-01
1.20e-01
8.00e-02
4.00e-02
0.00e+00
Cumulative Pore Volume (cc/g)
4.00e-02
3.66e-02
3.05e-02
2.44e-02
1.83e-02
1.22e-02
6.11e-03
0.00e+00
dV(d) (cc/nm/g)
3.000
5.000
10.000
20.000
30.000
40.000
300.000
Pore Diameter (nm)

HZ-V

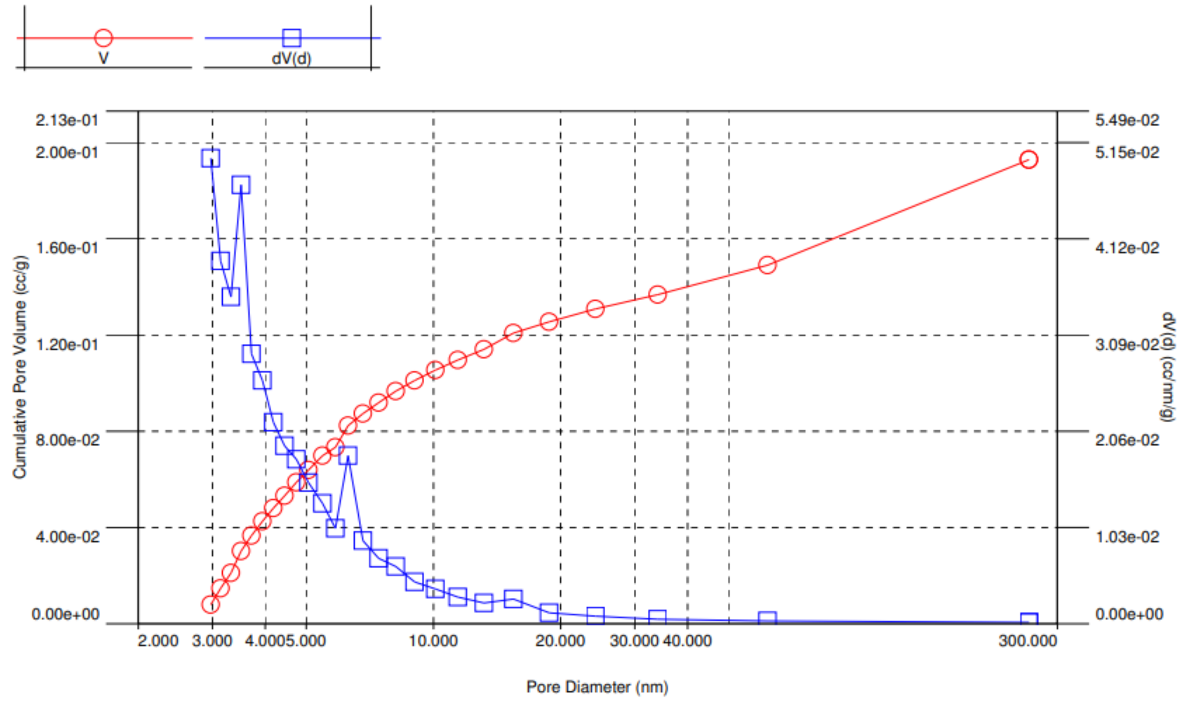

Figure S1. Pore size distribution of hierarchical materials.

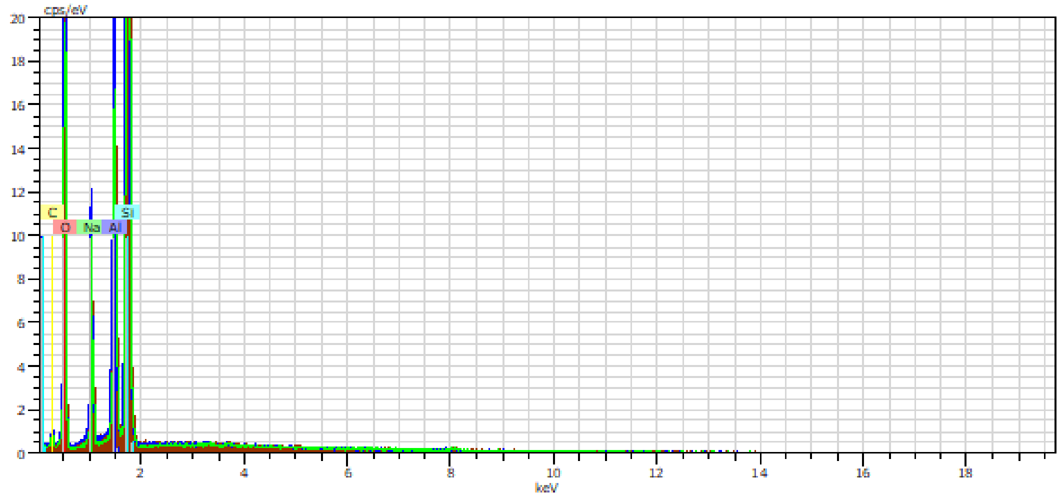

Mass percent (%)

Spectrum	C	O	Na	Al	Si
Mean value from 30 points:	3.34	42.36	1.79	13.07	30.43

Figure S2. EDX profile for HZ-commercial.

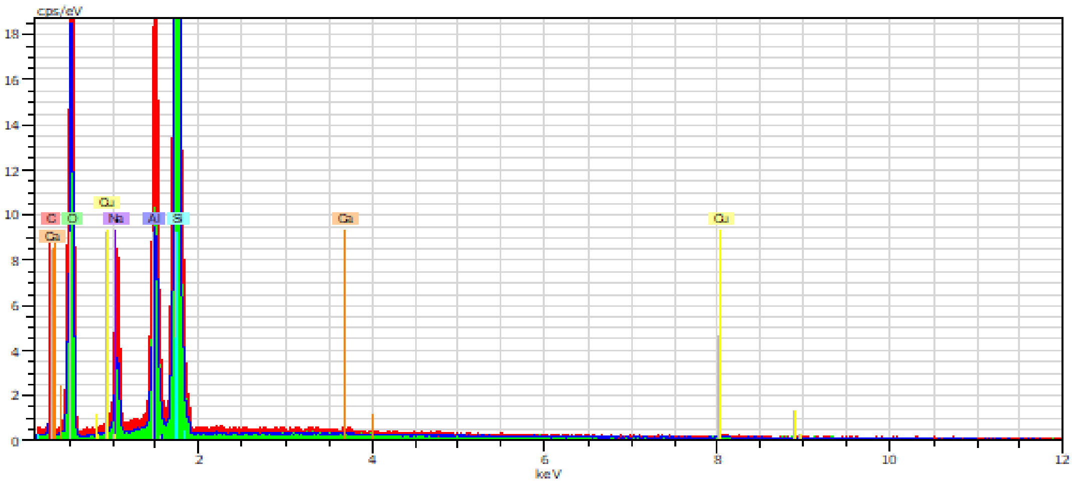

Mass percent (%)

Spectrum	C	O	Na	Al	Si	Ca*	Cu*
Mean value from 30 points:	2.36	47.07	6.10	9.70	34.12	0.14	0.50

* impurities

Figure S3. EDX profile for HZ.

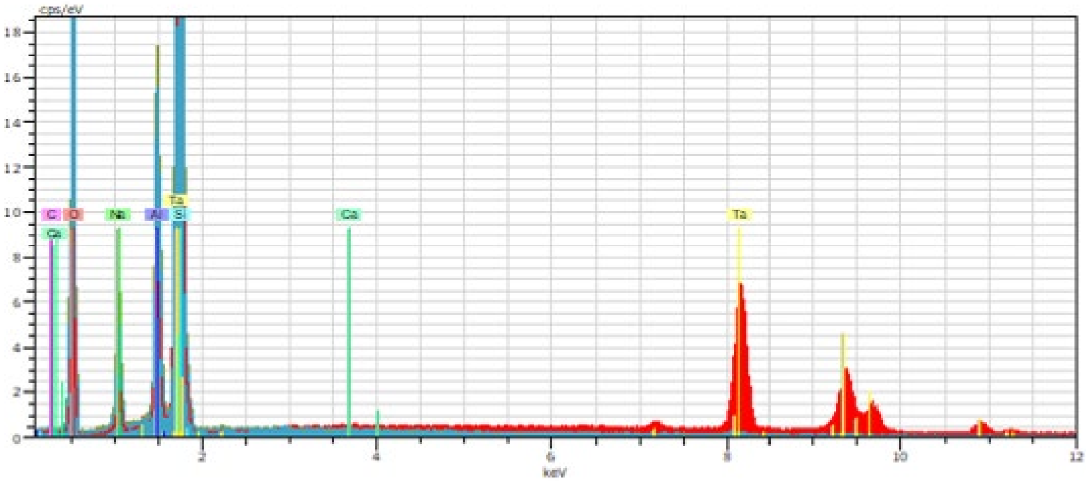

Mass percent (%)

Spectrum	C	O	Na	Al	Si	Ca*	Ta
Mean value from 30 points:	2.34	38.64	5.16	8.69	32.68	0.14	1.63

* impurities

Figure S4. EDX profile for HZ-Ta.

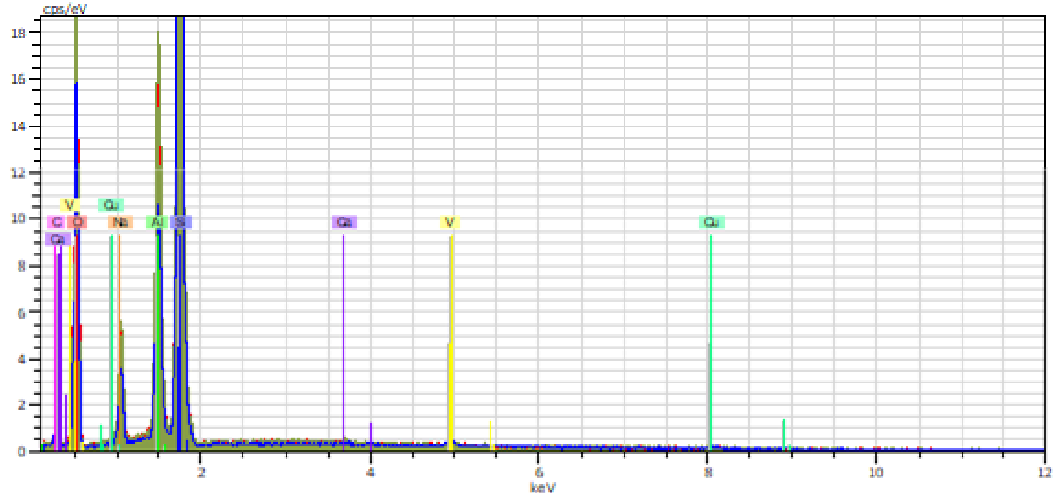

Mass percent (%)

Spectrum	C	O	Na	Al	Si	Ca*	V	Cu*
Mean value from 30 points:	3.24	42.95	5.75	10.66	36.51	0.05	0.35	0.49

* impurities

Figure S5. EDX profile for HZ-V.

Table S1. Chemical composition of zeolite Y (FAU)

SiO_2	57.94 wt.%
Al_2O_3	19.03 wt.%
Na_2O	1.95 wt.%
SiO_2/Al_2O_3 mole ratio	5.10
